# Theoretical and experimental study on the cooling time of a single device after sterilization based on a zero-dimensional heat transfer model

**DOI:** 10.1515/med-2026-1505

**Published:** 2026-09-02

**Authors:** Zhili Liu, Haiyi Yang, Xiaoxuan Chen, Huamei Ye, Yuewei Wang

**Affiliations:** Department of Nursing, Shantou University Mental Health Center, Shantou University Medical College—Faculty of Medicine of University of Manitoba Joint Laboratory of Biological Psychiatry, Third Clinical Institute of Shantou University Medical College, Shantou, Guangdong, China; Central Sterile Supply Department, Guangdong Provincial People’s Hospital (Guangdong Academy of Medical Sciences), Southern Medical University, Guangzhou, Guangdong, China; Central Sterile Supply Department, The First Affiliated Hospital of Shantou University Medical College, Shantou City, Guangdong Province, China

**Keywords:** autoclaving, cooling time, heat transfer, zero-dimensional model, central sterile supply department

## Abstract

**Objectives:**

To establish a zero-dimensional heat-transfer model for an isolated single-sealed 316 stainless-steel cylindrical device after pressure-steam sterilization and to evaluate whether the 30-min cooling time required by national standards provides an adequate safety margin under controlled conditions.

**Methods:**

Ambient conditions of the sterile-material storage area were used as boundary conditions. Biot number analysis was used to confirm applicability of the lumped-capacitance model, and the governing equation was derived from Newton’s law of cooling. Six 316 stainless-steel rods (diameter 4.5 mm, length 200 mm) were heated and cooled at 24 °C and 50 % relative humidity. Temperature was recorded every 5 s for 1,800 s, and each sample was tested three times. Cooling data were fitted with an exponential function in OriginPro.

**Results:**

The unadjusted theoretical model with h=5 W/(mˆ2 K) predicted 36.6 °C at 30 min, whereas the experimental fit approached ambient temperature and reached 24.6 °C. The fitted parameters were A=74.74 (95 % CI, 74.45–75.03), B=0.00402 sˆ-1 (95 % CI, 0.00399–0.00405), and C=24.56 °C (95 % CI, 24.53–24.59), with Rˆ2=0.9987 and a root mean squared residual of about 0.52 °C. The unadjusted theoretical curve is therefore a conservative reference rather than a point-accurate predictor.

**Conclusions:**

For the tested simplified single device, cooling followed an exponential pattern, reached body-temperature level within about 7–8 min, and approached room temperature within about 16–20 min. The 30-min rule provides an adequate margin for this bounded scenario, but the findings should not be generalized to complex instruments, packages, multiple loads, or variable airflow conditions without further validation.

## Introduction

Medical devices must be strictly sterilized before clinical use to ensure patient safety. Pressure steam sterilization has become the preferred clinical sterilization method because of its high efficiency, reliability, environmental protection and other advantages [1], [2], [3]. During autoclaving, the device is sterilized in a high temperature and high pressure steam environment (typically 134 °C, 205 kPa or 121 °C, 103 kPa) [4]. After sterilization, the device is at a high temperature and requires a proper cooling process for subsequent unloading, storage, and distribution.

The cooling process after sterilization is the key link in the whole sterilization process. If the cooling is not sufficient, the surface temperature of the device is too high, and when it comes into contact with relatively cold ambient air or packaging materials, it will cause the vapor to condense, forming the so-called “wet bag” phenomenon [5], 6]. Wet package refers to the sterilization package in which moisture and water droplets are visible inside or outside the package after autoclaving and proper cooling. Studies have shown that soaked sterile packaging materials can not be used as an effective bacterial barrier, which may lead to the contamination of sterile devices and make them unusable, which not only wastes resources, but also poses a potential threat to patient safety [7], 8].

There are differences between domestic and foreign standards on the cooling time of devices after sterilization. It is recommended to cool for 30 min after sterilization in China’s “Hospital Sterilization and Supply Center Part 2: Technical Operation Specification for Cleaning, Disinfection and Sterilization” (WS 310.2). The ANSI/AAMI ST79 standard recommends cooling the device to room temperature prior to subsequent handling, but does not specify a cooling time [9]. The European EN 285 standard also emphasizes the importance of cooling to room temperature, but the specific time is not clear enough [10]. This difference between standards brings confusion to the actual operation, and also reflects the lack of theoretical research on the cooling process of instruments.

Previous studies have explored the cooling of sterilized instruments mainly from empirical and operational perspectives. Dong Pengfei et al. [11] used infrared thermometry to monitor the cooling temperature of sterilized articles with different volumes and weights, showing that larger and heavier sterile packages required longer cooling. He Huiyan et al. [12] examined the influence of cooling time on the wet-pack rate and found that longer cooling was associated with a lower incidence of wet packs. Lv Sihang et al. [13] compared cooling methods for pressure-steam sterilization dressing bags and reported that ambient conditions affected cooling. These studies provide important clinical evidence, but most of them remain descriptive and do not explicitly link device cooling to heat-transfer parameters such as geometry, heat capacity, surface area, and convective heat-transfer coefficient.

In heat-transfer theory, the zero-dimensional heat-transfer model, also known as the lumped-capacitance method, is a classical approach for analyzing the cooling of small solid objects in air [14], 15]. This method assumes that the internal temperature of the object is approximately uniform and that the temperature varies only with time, thereby simplifying a three-dimensional unsteady heat-conduction problem into an ordinary differential equation. The method is applicable when the Biot number, Bi=hLc/λ, is less than 0.1, where h is the convective heat-transfer coefficient, Lc is the characteristic length, and λ is the thermal conductivity of the object [16]. Internationally, lumped-capacitance and related transient heat-transfer models have been used in metal processing, food cooling and drying, and heat dissipation of electronic devices [17], [18], [19], [20], [21]. These adjacent fields show that simplified heat-transfer models can be valuable for engineering estimation when their assumptions and boundaries are clearly stated.

However, direct application of a zero-dimensional heat-transfer model to post-sterilization cooling of medical devices has rarely been discussed in a systematic manner. For central sterile supply departments, the key practical questions are whether a 30-min cooling period is theoretically reasonable for a small isolated metal device, how the device temperature changes once it is taken out of the sterilizer, and how close the device temperature is to ambient and dew-point conditions during the cooling process. Addressing these questions may help transform an empirically adopted cooling time into a quantitatively interpretable operational reference.

The rationale of this study was that the post-sterilization cooling of a small, high-conductivity metal device may be approximated by a lumped-capacitance heat-transfer framework under controlled natural-convection conditions. The study hypothesis was that, for an isolated 316 stainless-steel cylindrical device with Bi<0.1, the temperature-time profile follows an exponential decay law and the 30-min cooling time provides a conservative safety margin. The research question was: under controlled ambient temperature and humidity, how well does a zero-dimensional heat-transfer model describe the cooling tendency of a simplified single medical device, and what practical boundary should be recognized when interpreting the 30-min cooling rule?

## Methods

### Symbol description

The symbols of the main physical quantities involved in this study and their meanings are as follows:

**Table:** 

h	Natural convection heat-transfer coefficient, W/(mˆ2 K)	ρ	Density, kg/mˆ3
V	Device volume, mˆ3	c	Specific heat capacity, J/(kg·K)
A	Device surface area, mˆ2	T0	Initial device temperature, K
λ	Thermal conductivity, W/(m·K)	T∞	Ambient air temperature, K
τ	Cooling time, s	θ	Excess temperature, K
Bi	Biot number, dimensionless	r	Device radius, m
L	Device length, m	d	Device diameter, m

### Model assumptions and applicability analysis

Single-piece single-seal instruments include small metal instruments such as scalpel handles, Kirschner wires, and orthopedic screws. These devices are characterized by relatively small volume, high thermal conductivity, and limited thermal mass. The present model focuses on the metal device as the dominant thermal body and treats the paper-plastic pouch and residual internal air as boundary elements rather than as additional heat-storage bodies. This assumption is reasonable for a simplified isolated-device analysis, but it does not imply that packaging is universally negligible in clinical practice. Packaging materials, pouch size, residual moisture, and the microclimate inside the pouch may influence local airflow, vapor transport, and condensation risk, and these factors define an important boundary for model interpretation.

According to heat-transfer theory, the zero-dimensional model can be used when the Biot number satisfies Bi=hLc/λ≤0.1, where Lc=V/A is the characteristic length, h is the convective heat-transfer coefficient, V is the device volume, A is the device surface area, and λ is the thermal conductivity of the device material [14], [15], [16]. For air natural convection, h is usually within 1–10 W/(mˆ2 K), although the effective coefficient may increase when weak airflow, support conduction, or radiative heat transfer contributes to heat loss [16].

A 316 stainless-steel cylindrical rod with a diameter of 4.5 mm and a length of 200 mm was selected as the representative simplified single device. According to the literature [22], the thermal conductivity of 316 stainless steel is λ=15.2 W/(m·K). For this cylinder, V=πrˆ2L=3.18 × 10ˆ-6 mˆ3, A=πdL + 2πrˆ2=2.86 × 10ˆ-3 mˆ2, and Lc=V/A=1.11 × 10ˆ-3 m. Using the conservative natural-convection coefficient h=5 W/(mˆ2 K), Bi=hLc/λ=3.66 × 10ˆ-4, which is far below 0.1. Therefore, the internal thermal resistance of this object is much smaller than the external heat-transfer resistance, and its internal temperature can be regarded as approximately uniform for the purposes of the present model.

The applicable scope of the zero-dimensional model is therefore limited by Biot number, geometry, material homogeneity, and boundary conditions. For a slender cylindrical device such as the sample used in this study, the small characteristic length makes the lumped-capacitance assumption appropriate. For forceps, scissors, hinged instruments, concave structures, or device assemblies with multiple contact points, the characteristic length and heat-transfer pathway may differ substantially. When the device size increases, when instruments are loaded together, or when packaging restricts airflow and moisture exchange, the model may no longer provide a point-accurate prediction and should be regarded only as a conservative screening framework unless additional validation is performed.

### Derivation of governing equations

Under the zero-dimensional assumption, the cooling process can be described by Newton’s law of cooling and the first law of thermodynamics. If the device temperature at time τ is T, the heat loss to the environment per unit time equals the decrease in internal energy of the device: −ρcV(dT/dτ) = hA(T – T∞), with the initial condition T(τ=0) = T0. Here, ρ is density, c is specific heat capacity, V is volume, h is the convective heat-transfer coefficient, A is surface area, T0 is the initial device temperature, T∞ is ambient air temperature, and τ is cooling time.

The excess temperature is defined as θ=T – T∞. After separation of variables and integration, ln(θ/θ0) = −hAτ/(ρcV), where θ0=T0 – T∞. The temperature equation is therefore T(τ) = (T0 – T∞)exp[-hAτ/(ρcV)] + T∞. This equation indicates that the device temperature approaches ambient temperature exponentially, and that the cooling rate is governed by hA/(ρcV), which represents the ratio of external heat-transfer capacity to device heat capacity.

### Calculation of theoretical cooling curve

According to the literature [22], [23], [24], [25] and differential scanning calorimetry in this study, the physical parameters of 316 stainless steel were as follows: density ρ=7,772 kg/mˆ3, specific heat capacity c=580 J/(kg·K), and thermal conductivity λ=15.2 W/(m·K). The environmental parameters of the sterile-goods storage area were based on WS 310.1–2016 [25], with ambient temperature in the range of 20–25 °C and relative humidity of 30 %–60 %. In this calculation, T∞=297 K (24 °C), T0=373 K (100 °C), and h=5 W/(mˆ2 K) were used for the unadjusted conservative theoretical curve.

The resulting theoretical equation was T(°C) = 76exp(-9.97 × 10ˆ-4τ) + 24. According to this model, the device cools from 100 °C to 60 °C in approximately 12.5 min, to 40 °C in approximately 26.0 min, to 37 °C in approximately 29.5 min, to 36.6 °C at 30 min, and to 26 °C in approximately 60.8 min. These estimates represent the conservative unadjusted natural-convection prediction and are not intended to replace local validation under different airflow, loading, or packaging conditions.

### Sensitivity analysis of physical property parameters

Because medical devices can be made of 316 stainless steel, 304 stainless steel, titanium alloy [26], cobalt-chromium alloy, and other materials, sensitivity analysis was performed to evaluate the influence of physical properties on the cooling curve. With the same simplified geometry and boundary conditions, the calculated temperature after 30 min varied modestly across common metallic materials because the product ρc and the thermal conductivity remained within ranges compatible with a small Biot number. Titanium alloy showed a faster cooling tendency because of its lower density and heat capacity, whereas cobalt-chromium alloy showed a slower tendency because of its higher heat capacity. This analysis supports the use of material properties in model-based estimation, but the results remain theoretical and require experimental confirmation for devices with different geometry, size, packaging, or contact conditions.

### Experimental materials

In this study, Fengji brand 316 stainless steel cylindrical rod (diameter 4.5 mm, length 200 mm) was used as a simulated single sealing device. The basis for selecting the device of this specification is as follows: firstly, 316 stainless steel is one of the most commonly used materials for medical devices, which is representative; secondly, the cylindrical rod of this specification has a regular geometric shape, which is convenient for theoretical calculation and experimental verification; thirdly, its size is similar to that of single-sealed devices such as Kirschner wire and orthopedic guide wire commonly used in clinic. There are 6 samples in total, numbered S1-S6.

The main instruments and equipment used in the experiment include: K-type thermocouple (Anhui Tiankang Group, model WRNK-191, thermometry range 0–400 °C, accuracy ± 0.5 °C, has been sent for calibration, calibration within the validity period); multi-channel temperature recorder (Yokogawa, Japan, model MV 2000, sampling accuracy 0.1 °C, sampling interval adjustable); Electronic balance (Mettler-Toledo, model ME204E, range 220 g, precision 0.1 mg, qualified by the annual verification of the Quality Supervision Bureau); differential scanning calorimeter (TA instrument, model DSC25, used to determine the specific heat capacity of the sample); pressure steam sterilizer (Shen’an Medical, model LDZX-50 KBS, used to heat the sample).

### Experimental environment

The experiment was carried out in the sterile storage area of the central sterile supply department. According to WS 310.1–2016, the temperature in this area is controlled at 20–25 °C and the relative humidity is 30 %–60 %. During the experiment, the environment was continuously monitored using a temperature-humidity recorder; the ambient temperature was 24 ± 1 °C (297 ± 1 K), relative humidity was 50 % ± 5 %, and atmospheric pressure was approximately 1 standard atmosphere. These controlled settings were used to isolate the cooling behavior of the simplified device and do not cover all airflow conditions encountered in routine hospital storage areas.

In order to ensure the natural convection conditions, the following measures were taken to control the experimental environment: the experiment was conducted in a relatively closed independent room, and the doors, windows and air conditioners of the room were closed during the experiment; the experimenters left the room after the samples were placed to avoid the airflow interference caused by the walking of the personnel; The sample was placed in a plexiglass isolation cover (size 40 × 30 × 30 cm), and the bottom of the isolation cover was provided with a ventilation gap to ensure natural convection and effectively shield the external airflow disturbance; the thermocouple lead was led out through the reserved hole of the isolation cover, and the thermometry instrument host was placed outside the isolation cover to reduce the impact of the instrument heat dissipation on the sample.

The natural-convection heat-transfer coefficient was determined by an inverse cooling method. A copper standard sample with known mass, surface area, volume, and initial temperature was placed in the same experimental environment, and its temperature-time curve was recorded during natural cooling. The slope of ln[(T – T∞)/(T0 – T∞)] vs. τ was obtained by least-squares fitting, and h was calculated from h=kρcV/A. The coefficient was measured three times, and the average value was h=5.0 ± 0.3 W/(mˆ2 K), which falls within the typical range for air natural convection [16]. Radiative heat transfer was not included in the main theoretical equation; instead, its potential contribution was considered in the model-boundary and error analysis because radiation and support conduction can increase the effective heat-transfer coefficient during the early high-temperature stage.

### Experimental method

The experimental process is as follows: Firstly, the specific heat capacity of the sample is measured by differential scanning calorimetry (DSC) according to ASTM E1269-11 (2018) standard. The test environment conditions are 23–25 °C temperature, 50–55 % relative humidity and 1standard atmospheric pressure. The density was measured by Archimedes drainage method, and the average value was obtained after three measurements.

**Figure 1:**
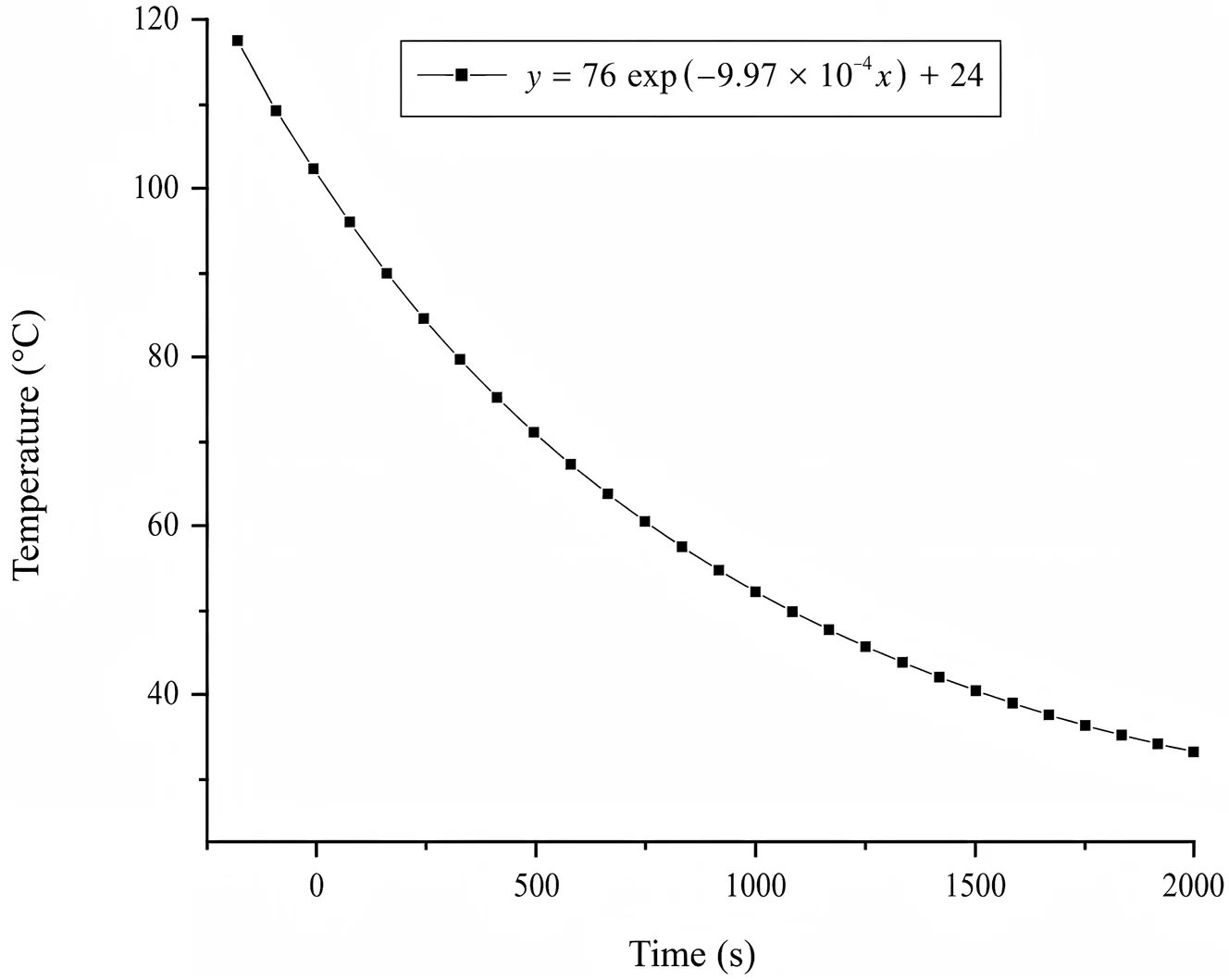
Unadjusted theoretical temperature curve for natural-convection cooling of an isolated single-piece device, plotted over 0–2,000 s for theoretical visualization.

The cooling experiment was performed as follows. Each sample was placed in a pressure steam sterilizer, heated to 120 ± 2 °C, and maintained for 30 min to ensure uniform temperature. The sample was then removed and placed rapidly in the isolation cover within the experimental environment. The K-type thermocouple probe was attached closely to the middle surface of the sample using high-temperature-resistant adhesive tape. When the sample temperature decreased to 100 °C (373 K), this time point was defined as 0 s. The temperature recorder continuously collected temperature data at 5-s intervals for 1,800 s. This 1,800-s duration refers to the experimental recording period; the 0–2,000 s horizontal range in Figure 1 is used only for displaying the unadjusted theoretical curve. Each of the six samples was tested three times, yielding 18 experimental cooling profiles. The experiment evaluated the simplified core-device cooling process; therefore, complete packaged-device cooling and multi-instrument loading were outside the direct experimental scope.

### Data processing and analysis

The experimental data were processed using OriginPro 2025a. Raw temperature-time data were imported, visually checked, and screened for obvious abnormal points defined as values deviating by more than three standard deviations from neighboring values. The three repeated measurements for each sample were averaged, and the average curves from the six samples were then combined to obtain the final experimental cooling profile. Exponential fitting was performed using the least-squares function T=A·exp(-Bτ) + C. Parameter estimates were reported with standard errors and approximate 95 % confidence intervals. Model performance was described using Rˆ2, the root mean squared residual, and the absolute and relative discrepancy between the unadjusted theoretical prediction and the experimental fitted temperature at key time points.

### Ethical approval and informed consent

This study used non-living stainless-steel samples and did not involve human participants, human tissue, personal data, or animal experiments. Ethical approval and informed consent were therefore not applicable.

## Results

### Measurement results of physical property parameters

The specific heat capacity of the sample determined by differential scanning calorimetry was c=580 ± 8 J/(kg·K), and the density determined by Archimedes’ method was ρ=7,772 ± 15 kg/mˆ3. Physical parameters of common medical device materials are shown in Table 1.

**Table 1:** Physical parameters of common medical device materials.

Material	ρ (kg/m˄3)	c (J/(kg·K))	λ (W/(m·K))
316 stainless steel	7,772	580	15.2
304 stainless steel	7,930	500	16.2
Ti-6Al-4V titanium alloy	4,430	526	6.7
Co-Cr alloy	8,300	450	14.8

### Theoretical cooling curve

Substituting the physical parameters into the temperature equation produced the theoretical cooling curve shown in Figure 1. The unadjusted theoretical model with h=5 W/(mˆ2 K) predicted that the temperature would decrease from 100 °C to 36.6 °C after 30 min of cooling. The horizontal axis in Figure 1 extends to 2,000 s to show the theoretical curve more completely, whereas the experimental temperature recording lasted 1,800 s.

### Cooling test results

**Figure 2:**
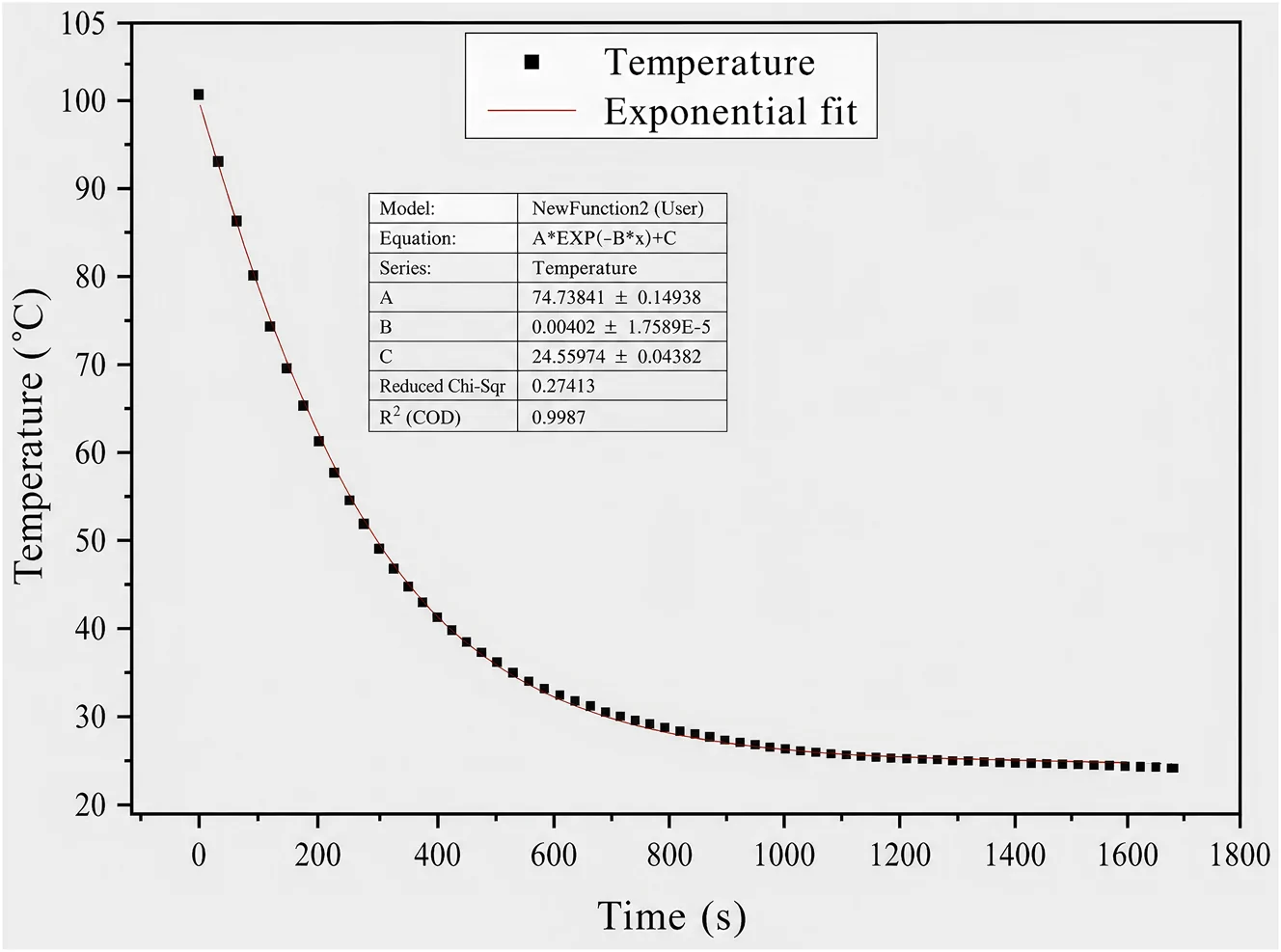
Experimental fitted temperature curve for natural-convection cooling of an isolated single-piece device.

Figure 2 shows the experimental cooling profile and exponential fitting results for the simplified single-device cooling experiment. The fitted equation was T(°C) = 74.74exp(-0.00402τ) + 24.56. The parameter estimates were A=74.74 ± 0.15, B=0.00402 ± 0.000015 sˆ-1, and C=24.56 ± 0.014 °C, corresponding to approximate 95 % confidence intervals of 74.45–75.03, 0.00399–0.00405 sˆ-1, and 24.53–24.59 °C, respectively. The coefficient of determination was Rˆ2=0.9987, and the root mean squared residual was approximately 0.52 °C, indicating that the exponential decay function closely described the observed cooling trend.

### Comparison between theoretical and experimental values

Table 2 compares the unadjusted theoretical temperatures with the experimental fitted temperatures at key time points. The experimental cooling profile was faster than the theoretical prediction based on h=5 W/(mˆ2 K), especially during the early and middle cooling stages. At 30 min, the theoretical temperature was 36.6 °C, whereas the experimental fitted temperature was 24.6 °C. The mean absolute discrepancy across the four selected time points was 25.4 °C, and the root mean squared discrepancy was 27.1 °C. Therefore, the theoretical model was used to describe the exponential form and conservative tendency of cooling rather than to claim exact point-by-point prediction under all boundary conditions.

**Table 2:** Comparison between theoretical prediction and experimental fitted temperature.

Time, min	Theoretical T, °C	Experimental fitted T, °C	Difference, °C
5	80.4	46.9	33.4
10	65.8	31.3	34.5
20	47.0	25.2	21.8
30	36.6	24.6	12.0

### Analysis of key time nodes

According to the experimental fitted profile, the device cooled to body-temperature level (about 37 °C) after approximately 7–8 min and approached 26 °C after approximately 16–17 min. At 30 min, the fitted temperature was 24.6 °C, which was close to the ambient room temperature. These findings indicate that the 30-min cooling time recommended by the national standard provides a substantial margin for this simplified isolated stainless-steel device, while the margin for complex packages or multi-instrument loads must be evaluated separately.

### Reference table of cooling time

Based on the theoretical framework and the experimentally inferred effective cooling coefficient, an illustrative cooling-time reference table was compiled for selected simplified single-device scenarios (Table 3). The table is intended for model interpretation and process discussion rather than as a universal release criterion.

**Table 3:** Illustrative cooling-time reference for simplified isolated single-device scenarios (ambient temperature 24 °C).

Device type	Material	Feature size	To 40 °C	To 35 °C	To ∼26 °C
Kirschner wire	316 stainless steel	φ4.5 mm isolated cylinder	∼6–7 min	∼8 min	∼15–16 min
Orthopedic screw	Ti-6Al-4V titanium alloy	φ6 mm simplified cylinder	∼4–5 min	∼5–6 min	∼10 min
Small pliers	Stainless steel	5 mm simple geometry	∼7–8 min	∼9–10 min	∼17–20 min

Values are model-based estimates for isolated single-device scenarios using the experimentally inferred effective cooling coefficient. They are not release criteria and should be validated locally for complex instruments, packaging, airflow, and multi-instrument loads.

## Discussion

### Model validation and error analysis

The research question of this study was whether a zero-dimensional heat-transfer model could describe the cooling tendency of an isolated simple stainless-steel device after sterilization and help interpret the 30-min cooling rule. The findings answer this question by showing that the device cooling profile followed an exponential decay pattern and that the device temperature approached ambient temperature within the 30-min standard under controlled conditions. However, the unadjusted theoretical prediction using h=5 W/(mˆ2 K) was not point-accurate at later and selected early time points; it should be interpreted as a conservative engineering estimate. The experimental attenuation coefficient B=0.00402 sˆ-1 corresponds to an effective heat-transfer coefficient of approximately 20.2 W/(mˆ2 K), which is higher than the conservative natural-convection value. This difference is plausibly related to weak residual airflow, heat conduction through the support, radiative heat loss during the high-temperature stage, and surface-probe contact conditions. The validation of the model in this study therefore concerns the applicability of the exponential cooling structure and the adequacy of the 30-min margin for the tested simplified scenario, not universal point prediction for all sterilization workflows.

### Comparison with existing studies

The results are broadly consistent with previous clinical observations that smaller and lighter sterilized articles cool more rapidly than larger packages [11], [12], [13]. Compared with these empirical studies, the present work links cooling time to heat capacity, surface area, heat-transfer coefficient, and Biot number. Similar lumped-capacitance or simplified transient heat-transfer concepts have been applied in fields such as food cooling, wood drying, and electronic heat dissipation [19], [20], [21], where model usefulness depends on clearly defined boundary conditions and validation against representative experiments. This cross-disciplinary evidence supports the theoretical rationale for using a zero-dimensional framework as a first-order estimate, while also emphasizing that application to medical devices should remain scenario-specific.

### Discussion on applicable boundary of the model

The zero-dimensional heat-transfer model in this study is mainly applicable to an isolated device that satisfies Bi<0.1, has a relatively simple geometry, and is exposed to a defined ambient environment. Actual surgical instruments may contain hinges, teeth, cavities, contact surfaces, and irregular three-dimensional shapes that alter both heat capacity and the effective surface area. In routine sterile processing, instruments are also frequently cooled together, placed in trays, wrapped, stored, or transported with other materials. These conditions increase the overall thermal mass and may reduce local airflow, thereby slowing cooling compared with the isolated rod tested here. Consequently, the model should not be generalized to large orthopedic sets, complex instruments, densely loaded trays, or overnight storage scenarios without additional experimental verification.

### Discussion on the formation mechanism of wet pack

Wet-pack formation is a moisture-condensation process involving the device surface, packaging material, residual vapor, and the microclimate within or around the package. In the present experimental environment of 24 °C and 50 % relative humidity, the estimated dew-point temperature was approximately 13 °C [27]. The experimental fitted device temperature was approximately 24.6 °C at 30 min, leaving an estimated margin of about 11 °C above the ambient dew point. Even at 60 % relative humidity, the dew point at 24 °C is approximately 15.7 °C, which remains below the measured 30-min temperature. This quantitative comparison indicates that ambient-air condensation on the device surface alone is unlikely under the tested conditions. Nevertheless, wet packs can also occur when residual moisture, insufficient drying, cooler packaging materials, or restricted vapor exchange creates a local microclimate inside the pouch. Cooling time is therefore only one factor in wet-pack prevention and should be considered together with drying performance, packaging integrity, storage ventilation, and humidity control.

### Recommendations for practical application

For practical operation in a central sterile supply department, the present findings support the 30-min cooling time for small isolated single-piece metal devices under controlled ambient conditions. The conclusion should be applied conservatively. For instruments with complex geometry, higher thermal mass, dense tray loading, paper-plastic pouch constraints, or exposure to active air circulation, local temperature monitoring or validation is recommended before shortening routine cooling procedures. Modern sterilization practice also includes adequate drying cycles, ventilated clean storage areas, avoidance of contact with cold surfaces, and standardized handling procedures; these practices interact with cooling time to reduce wet-pack risk [28], 29]. The model can help staff understand temperature change and safety margin, but it should not be used as the sole determinant of release decisions.

### Study limitations

This study has several limitations. The experimental object was a simplified 316 stainless-steel cylindrical rod, and clinically representative instruments with complex geometry were not tested. The experiment was conducted under a single controlled temperature and humidity condition, and it did not systematically examine active airflow, ventilated storage, operational disturbance, extended cooling for several hours or overnight, or repeated handling. Complete paper-plastic packaging, residual moisture, and multi-instrument loading were not experimentally modeled, although these factors are directly relevant to real-world wet-pack formation. The theoretical model also did not explicitly include radiative heat transfer, contact conduction, or coupled heat-and-mass transfer inside a pouch. These limitations restrict the generalizability of the findings and define the need for further validation.

### Future research directions

Future studies should include representative medical instruments with different geometries, materials, and loading patterns; compare exposed devices with packaged devices; and assess the effects of ambient temperature, humidity, airflow, and storage ventilation on both temperature decline and wet-pack incidence. Additional models should incorporate humidity, dew point, packaging permeability, and residual moisture to establish a coupled heat-and-mass-transfer framework. Portable temperature and humidity monitoring could be combined with decision-support tools to guide process validation in central sterile supply departments. In this context, recent medical research on large language models and machine-learning-assisted evidence synthesis highlights both the potential and the concerns of artificial-intelligence tools in medicine, including accuracy, safety, bias, and governance [30]. Such tools may assist literature surveillance and protocol support in the future, but any operational recommendations generated from them should be validated by local experimental and clinical evidence.

## Conclusions

In this study, a zero-dimensional heat-transfer model was applied to the post-sterilization cooling of a simplified single-piece stainless-steel device. The derived exponential temperature equation was consistent with the observed exponential cooling pattern, and the experimental fit showed high goodness of fit. Under controlled ambient conditions of 24 °C and 50 % relative humidity, the tested device cooled to approximately body-temperature level within about 7–8 min and approached room temperature within about 16–20 min; at 30 min, the fitted temperature was close to the ambient temperature. These findings support the adequacy of the 30-min cooling standard for the tested isolated simple metal device. Because the unadjusted theoretical model was conservative and the experiment did not include complex instruments, multi-instrument loads, active airflow, or full packaging effects, the results should be interpreted as a bounded theoretical and experimental reference rather than a universal prediction for all clinical sterilization workflows.
